# Shyness in Early Infancy: Approach-Avoidance Conflicts in Temperament and Hypersensitivity to Eyes during Initial Gazes to Faces

**DOI:** 10.1371/journal.pone.0065476

**Published:** 2013-06-05

**Authors:** Yoshi-Taka Matsuda, Kazuo Okanoya, Masako Myowa-Yamakoshi

**Affiliations:** 1 Okanoya Emotional Information Project, Exploratory Research for Advanced Technology (ERATO), Japan Science and Technology Agency (JST), Saitama, Japan; 2 Emotional Information Joint Research Laboratory, RIKEN Brain Science Institute, Saitama, Japan; 3 Center for Baby Science, Doshisha University, Kyoto, Japan; 4 Department of Life Sciences, Graduate School of Arts and Sciences, The University of Tokyo, Tokyo, Japan; 5 Graduate School of Education, Kyoto University, Kyoto, Japan; University of Leicester, United Kingdom

## Abstract

‘Infant shyness’, in which infants react shyly to adult strangers, presents during the third quarter of the first year. Researchers claim that shy children over the age of three years are experiencing approach-avoidance conflicts. Counter-intuitively, shy children do not avoid the eyes when scanning faces; rather, they spend more time looking at the eye region than non-shy children do. It is currently unknown whether young infants show this conflicted shyness and its corresponding characteristic pattern of face scanning. Here, using infant behavioral questionnaires and an eye-tracking system, we found that highly shy infants had high scores for both approach and fear temperaments (i.e., approach-avoidance conflict) and that they showed longer dwell times in the eye regions than less shy infants during their initial fixations to facial stimuli. This initial hypersensitivity to the eyes was independent of whether the viewed faces were of their mothers or strangers. Moreover, highly shy infants preferred strangers with an averted gaze and face to strangers with a directed gaze and face. This initial scanning of the eye region and the overall preference for averted gaze faces were not explained solely by the infants’ age or temperament (i.e., approach or fear). We suggest that infant shyness involves a conflict in temperament between the desire to approach and the fear of strangers, and this conflict is the psychological mechanism underlying infants’ characteristic behavior in face scanning.

## Introduction

During the third quarter of the first year, many infants start reacting shyly to adult strangers, which is a behavior known as infant shyness [1]–[9]. Infant shyness is an early developing form of shyness that is induced by strangers and is distinct from later-developing forms of shyness, such as self-conscious shyness, that appear at approximately 4 or 5 years of age [10]. The definitions and/or criteria for infant shyness vary depending among studies and include, for example, inhibited approach, low sociability or a fear of strangers. Shyness and fear temperaments are conceptually similar in that both promote inhibition or withdrawal. A longitudinal study showed that parent-reported shyness correlated with the degree of fear at 18 months of age and that this relationship weakened at 30 months of age [11]. This study indicates that infant shyness can be explained by a fear temperament to some extent, but that shyness and fear temperaments are fundamentally different. One characteristic difference between infant shyness and fear is the respective relationships to approach. Fear is a separate temperament from approach behavior [12], whereas shyness seems to relate to both approach behavior and fear. Indeed, shy children may possess high avoidance tendencies that are induced by social fear and high approach tendencies. That is, although shy children desire social interaction, their approach-motivation is simultaneously inhibited by a competing avoidance-motivation, which is triggered by social fear and anxiety, i.e., conflicted shyness [13]–[17]. This approach-avoidance conflicted model of shyness has been adapted for shy children over 3 years of age but has not been studied in young infants, even though it may explain infant shyness, given that 4-month-old infants occasionally show positive shyness by coyly smiling at adult strangers [18]. This coy smile may reflect a feeling of ambivalence between pleasure and aversion during the social interaction [19]. Thus, shy infants may possess the approach-avoidance conflict that is observed in shy children. One aim of our study was to investigate whether the conflicted model of shyness can explain infant shyness.

Our second purpose was to investigate face scanning in shy infants. One characteristic of shy behavior is the avoidance of eye contact [20]–[25]. However, few studies have used eye tracking to reliably capture precise face scanning patterns in relation to shyness. Brunet et al. [26] investigated 11-year-old shy children and found that shyness was associated with longer dwell times in the eye region than in the mouth region, which suggests that some shy children are not avoiding the eyes (at least in a laboratory setting). We wondered whether highly shy infants also increase their time spent looking at the eyes when compared with less shy infants. We further questioned whether shy infants scan the faces and facial parts of their mothers and strangers differently from non-shy infants, given that shy behavior is typically evident with strangers but not with familiar individuals in a cue-dependent manner. We also investigated how face/gaze direction affects face preferences in shy infants as an index of face-to-face contact with strangers. Given that shyness is characterized as a tendency to escape from social interaction with strangers [10], [27], shy infants may prefer the averted gaze/face of strangers to the direct gaze/face.

To investigate these questions, we recruited infants across a range of ages (7 to 13 months old; m.o.) because the timing of the appearance and the strength of infant shyness vary [5]. The Colorado Child Temperament Inventory (CCTI) [10], [27] was used to assess the degree of shyness for each infant, and fear and approach temperament characteristics were measured by the Infant Behavior Questionnaire Revised (IBQ-R) [12], based on scores from maternal reports. A preferential-looking paradigm was used to investigate infants’ face scanning by presenting a pair of faces side-by-side on an eye-tracking screen. We presented the following three types of face stimuli: mothers, strangers and faces that are intermediately between mothers and strangers. Intermediate faces were created using a morphing technique with a physical composition of 50 percent of the mother’s face and 50 percent of the stranger’s face. Previous studies have demonstrated that infants spend *less* time looking at these hybrid faces than at either their mother or a stranger’s face [28]. We used the intermediate faces to assess how shyness affects infants’ sensitivity to their mothers’ faces. If shy infants are sufficiently sensitive to their mothers’ faces, they should prefer their mothers’ faces to the intermediate faces (as observed in normal infants). Furthermore, we expected that shy infants may prefer intermediate faces to strangers’ faces despite the imperfectness of the hybrid pseudo-mothers’ faces, which would differ from the preference of less-shy infants.

## Materials and Methods

### Ethics Statement

The participants’ parents provided written informed consent and the Behavioral and Social Science Ethical Review Committee of Kyoto University specifically approved this study (Application #20090901). Subjects presented in the photographs in figures provided written informed consent, as outlined in the PLOS consent form, regarding the publication of their photographs.

### Participants

We recruited infants from a wide range of ages (7–13 m.o., average of 9.8 m.o., SD = 1.9) because the appearance of shy behaviors in infancy varies from individual to individual [5]. Fifty-seven infants (23 male, 34 female; ages 7.0 to 13.3 m.o.) and their mothers were invited to visit the lab twice. On the first day, the mothers’ photographs were taken for use as visual stimuli. The experiments were conducted approximately 1 week after the first visit. Six additional infants were excluded from the experimental analysis because they did not complete the eye-tracking protocol.

### Measures: Parent Questionnaires

Both the CCTI and IBQ-R questionnaires were provided to mothers during their second visit to the laboratory. The mothers were asked to answer each item about their infants’ behavior with regard to the past seven days.

CCTI [10], [27]: The shyness scale from the CCTI consists of the following five items: “My child takes a long time to warm up to strangers”, “My child tends to be shy”, “My child makes friends easily (reversed)”, “My child is very friendly with strangers (reversed)” and “My child is very sociable (reversed)”. Each item was scored from 1 (not at all) to 5 (very much). The scores were summed across the 5 items for each infant was as a shyness score, which had 5 as the minimum score, 25 as the maximum score and 15 as the intermediate score. Although the CCTI was designed for children aged 1–6 years [10], [27], we adapted the shyness questionnaire for our current sample of 7- to 13-month-old infants given that the inter-individual variation of scores (i.e., standard deviations) and the internal consistency of the shyness scale (i.e., the Cronbach’s alpha) were consistent with those of a published sample [27]. The standard deviations were 4.6 for our sample and 5.1 for the published sample, and the Cronbach’s alpha was 0.85 for our sample and 0.88 for the published sample. In the face preference experiments, we divided infants into two subgroups based on their CCTI scores: infants with high (score >15; intermediate score) and low (score ≤15) shyness. Age did not differ between the two subgroups (t_31_ = 1.98, P>0.05) (mean = 10.63 m.o., SD = 1.86, N = 17 in the high shyness subgroup) (mean = 9.56 m.o., SD = 1.78, N = 34 in the low shyness subgroup).

IBQ-R [12]: We also asked mothers to answer the items from the *Fear* and *Approach* scales of the IBQ-R, which is a questionnaire designed for infants in the first year of life and is suitable for use with our sample. The fear scale consisted of 16 questions regarding both social and non-social contexts (e.g., “When your baby was approached by an unfamiliar person when you and s/he were out, how often did the baby cry?” and “When visiting a new place, how often did the baby continue to be upset for 10 minutes or more?”). The approach scale consisted of 12 questions regarding both social and non-social contexts (e.g., “When familiar relatives/friends visited, how often did the baby get excited?” and “When given a new toy, how often did the baby get very excited about getting it?”). Each item was scored from 1 (not at all) to 7 (very much), and the average score was calculated for each scale. The values of Cronbach’s alpha for the *Fear* and *Approach* scales of the IBQ-R for the present sample were 0.88 and 0.82, respectively, and were generally similar to the values reported by Garstein and Rothbart (2003) [12].

In the face preference experiments, we divided infants into two subgroups based on their IBQ-R scores as follows: infants with high (score ≥4; intermediate score) and low (score <4) fear, infants with and high (score ≥5) and low (score <5) approach. We adopted the borderline score of ‘5’ for the approach temperament based on the average value for all of the infants as opposed to taking the intermediate score of ‘4’, given that the lower score imbalanced the number of infants categorized as having high (48 infants) and low (3 infants) approach scores. In the two subgroups for fear temperament, age was significantly different between the subgroups (t_36_ = 2.79, P<0.01) (mean = 10.81 m.o., SD = 1.80, N = 19 in the high fear subgroup) (mean = 9.39 m.o., SD = 1.71, N = 32 in the low fear subgroup). Age did not differ between the high and low approach subgroups (t_32_ = 0.77, P>0.77) (mean = 9.86 m.o., SD = 1.80, N = 33 in the high approach subgroup) (mean = 10.03 m.o., SD = 2.01, N = 18 in the low approach subgroup).

In our current study, *temperament* refers to the individual personality differences in infants and young children that are present prior to the development of more sophisticated cognitive and social aspects of personality [29], whereas *trait* refers to a more mature form of personality differences and habitual patterns of behavior, thought and emotion that is are relatively stable over time [30].

### Apparatus

A Tobii (Stockholm, Sweden) X60 Eye Tracker was used to record the infants’ looking behavior. The eye tracker was integrated with a 23-inch LCD monitor that displayed the stimuli using Tobii Studio AVI presentation software. Infants were seated on a parent’s lap approximately 60 cm from the monitor that presented the stimuli. During the experiment, parents were asked to look below the monitor to avoid influencing which stimulus their infant looked at. A video camera was placed near the top of the screen, through which the experimenter monitored the infant’s face. A five-point calibration was administered before the recording (for technical details about the apparatus and the calibration procedure, see [31], [32]).

### Visual Stimuli

Color photographs of the mothers and female strangers were taken prior to the experiments. Images of smiling and neutral faces with both direct and averted head/gaze postures (i.e., faces looking toward or away from the subjects) were taken for each individual. The photographs showed a face with the individual’s hair pinned up and the individual’s face without glasses. Rather than using still images of smiling faces, we created movie stimuli for both the mothers and strangers, which were termed dynamic facial expressions [33] because infants are more responsive to moving faces than to static faces [34]. Movie stimuli were created in the following manner. Using the neutral and smiling expressions for each person, 24 intermediate images in 4% steps were created using computer-morphing techniques (Sqirlz Morph 2.1: Xiberpix, Solihul, UK, www.xiberpix.com). To create a moving clip, 26 images (i.e., 1 neutral image, 24 intermediate images and the final smiling image) were presented in succession. Each image was presented for 40 ms, and the first and last images were additionally presented for 480 ms. Thus, each animation clip lasted for 2,000 ms. Each clip was repeated 5 times (i.e., for a 10-second duration) during the main experiments. For adults, this presentation speed sufficiently reflects natural changes in the dynamic facial expressions of happiness [33].

To create the intermediate faces, the faces of a mother and a stranger were morphed together to produce a new face that consisted of 50% of the mother’s face and 50% of the stranger’s face [28]. Then, movie stimuli for the dynamic facial expressions were created using the neutral and smiling expressions for each intermediate face according to the procedure outlined previously.

### Procedure

The infants saw the following three pairs of stimuli: mother *vs.* stranger, mother *vs.* intermediate face and stranger *vs.* intermediate face. The presentation was repeated twice with photographs of different strangers used as the stimuli representing the strangers and intermediate faces. Each face stimulus subtended a visual angle of 11.13° × 12.50° from a distance of 60 cm. Each test trial was presented for 10 seconds. Each trial was preceded by a stimulus that was intended to attract the infants’ visual attention. The order of the six test trials and the side that a given face appeared were random and counterbalanced across participants. A mother’s face was used as the stranger’s face for the other participants to furnish a homogeneous set of stimuli in this study. After the experiment, we confirmed with each mother that the strangers whose faces were presented were not acquaintances of her infant.

The total time spent looking at each stimulus type was averaged across all of the test trials for each individual and then normalized to calculate proportions. The proportions were transformed with the arcsine function to achieve a normal distribution.

## Results

### Infants’ Shyness Scores

Shyness scores for the infants in the second half of the first year (N = 57) are depicted as a function of the infants’ ages in Figure S1. No significant correlation was found between the shyness scores and age (R = 0.18, t_55_ = 1.36, P>0.10), which indicates that there was large individual variation. No significant differences in gender were found for the shyness score (t_55_ = 0.12, P>0.90, Cohen’s *d* = 0.03).

### Relationships between Shyness Scores and Fear and Approach Scores

We also investigated the fear and approach scores for the same subjects. The fear scores showed a subtle but significant positive correlation with the infants’ age in the second half of the first year (R = 0.32, t_55_ = 2.50 P<0.05), whereas the approach scores did not show a significant correlation with age during this period (R = 0.09, t_55_ = 0.67, P>0.4). These results are consistent with previous reports that approach motivation appears very early in development and stays stable over time, whereas fear does not emerge until later developmental stages, specifically around the third quarter of the first year [5], [8], [35], [36].

**Figure 1:**
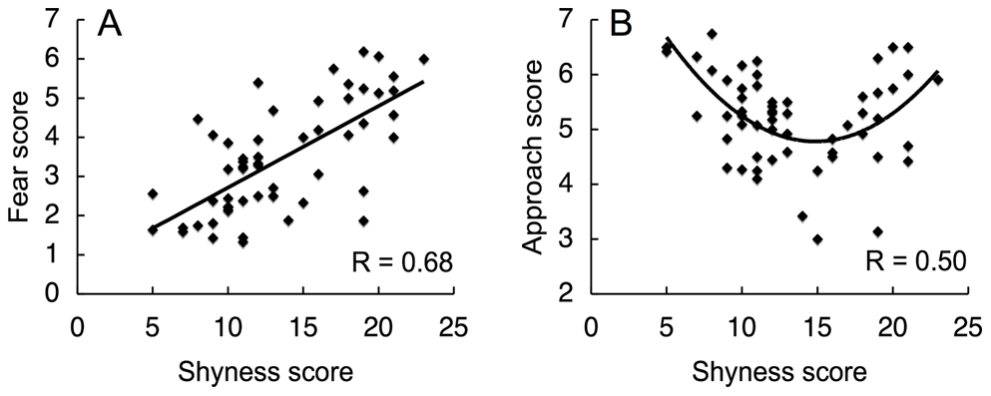
Relationship between shyness and other temperaments. (A) Fear scores (vertical axis) correlate linearly with shyness scores (horizontal axis). The solid line represents a regression line of the distribution. (B) Approach scores (vertical axis) correlate secondarily with shyness scores (horizontal axis). The solid line represents a secondary regression curve of the distribution. R: Correlation coefficient.

We then compared the shyness scores with the fear and approach scales. The shyness scores were significantly correlated with the fear scores (R = 0.69, t_55_ = 7.07, P<0.001, Fig. 1a), which may reflect similarity in the questionnaire items between the CCTI shyness scale and the IBQ-R fear scales with regard to social contexts [10], [12], [27]. However, the shyness scores also showed a significant secondary correlation with the approach scores (R = 0.50, t_55_ = 4.28, P<0.001, Fig. 1a), which indicates that both extremely high- and low-scoring shy infants had high approach scores. Notably, the fear and approach scores had a significant but modest correlation (linear correlation, R = 0.28, t_55_ = 2.16, P<0.05; secondary correlation, R = 0.28, t_55_ = 2.16, P<0.05), which indicates that they are independent of each other [12]. Taken together, the results of these questionnaire experiments reveal that highly shy infants possess conflicted temperaments with both high fear and high approach behaviors, as observed in shy children who are experiencing the approach-avoidance conflict [13]–[17].

### Face Preferences for Familiar and Novel Individuals

We next examined shy infants’ preferences for looking at the faces of familiar and novel persons using the eye-tracking system. We divided and classified the subjects on the basis of their shyness scores, irrespective of their age (Figure S1), into groups with high (score >15, N = 17) and low (score ≤ 15, N = 34) shyness.

**Figure 2:**
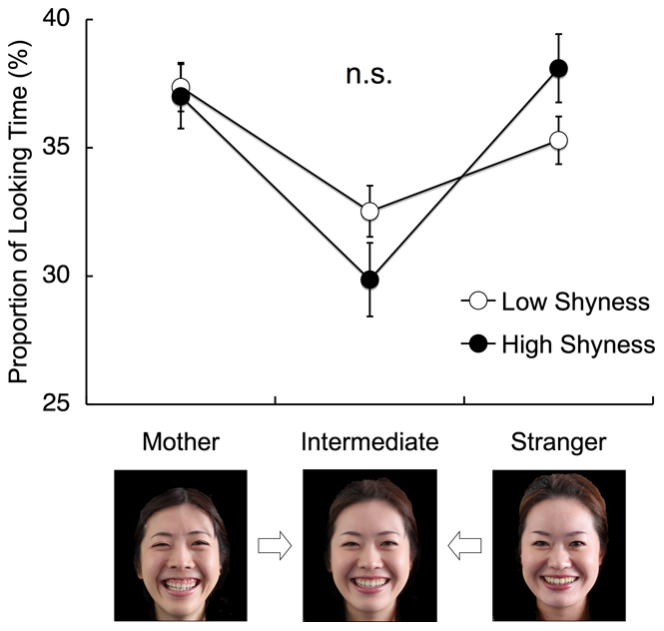
Infants’ visual preferences for different face types. This figure shows the mean percentile fixation durations for the following three face stimuli: mother, intermediate and stranger. The bottom pictures present examples of the face stimuli. The open and filled circles represent the mean fixation durations for the infants with low and high shyness, respectively. Error bars indicate the S.E. of the mean. n.s.: no significant difference.

Given that infants generally prefer both familiarity and novelty in objects [37], shy infants may be expected to show a preference for familiar persons (e.g., caregivers) over strangers. However, our results show that both the highly shy infants (>15 score in shyness) and the low-scoring infants (≤ 15 score) looked at the mothers and strangers’ faces for equal durations. Indeed, a 2 (shyness: high, low) × 2 (object: mother, stranger) repeated-measures analysis of variance (ANOVA) showed that there was neither a significant main effect of object (F_1,98_ = 0.19, P = 0.67, η_p_^2^<0.01) nor an interaction between shyness and object (F_1,98_ = 1.94, P = 0.17, η_p_^2^ = 0.02) (Fig. 2).

The equal preference of the infants to look at their mother’s and a stranger’s face indicates that infants’ looking time did not certify their ability to discriminate facial stimuli. By presenting faces that are intermediately between mothers and strangers [28], we confirmed that the infants indeed discriminated the stimulus faces. The intermediate faces were created using a morphing technique to synthesize a new face that consisted of 50% of the mother’s face and 50% of the stranger’s face (see Methods). Infants spend less time looking at the intermediate faces if they recognize the facial stimuli adequately [28]. Indeed, both the high and low shyness groups showed a significantly lower preference for the intermediate faces relative to both the mothers and strangers’ faces. A 2 (shyness: high, low) × 3 (object: mother, intermediate, stranger) repeated-measures ANOVA revealed a significant main effect of object (F_2,147_ = 16.21, P<0.001, η_p_^2^ = 0.18) and no significant interaction between shyness and object (F_2,147_ = 2.76, P = 0.07, η_p_^2^ = 0.04) (Tables S1 and S2). *Post-hoc* testing (Bonferroni) showed that infants preferred intermediate faces less than the faces of mothers (P = 0.001) and strangers (P = 0.009). These results indicate that, when the infants recognized that the strangers were novel, both the highly shy infants and the low-scoring infants increased their time spent looking at the strangers.

There were no significant differences in face preference with respect to the infants’ age, fear temperament and approach temperament (ANOVA and correlational analysis, Table S3).

### Different Facial Scanning Patterns

**Figure 3:**
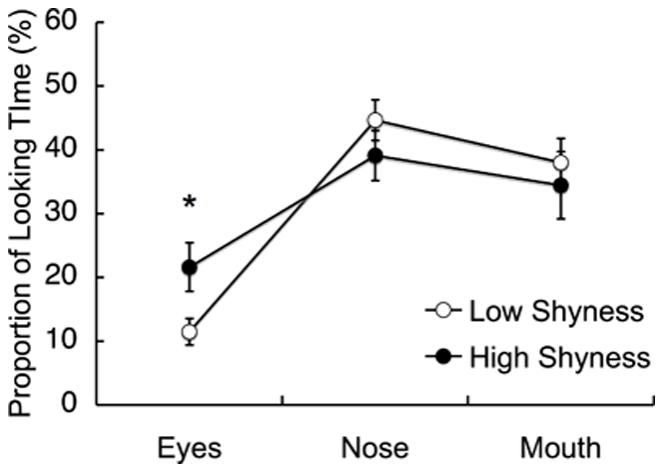
Infants’ visual preferences for different facial regions. This figure shows the mean percentile fixation durations for the following three types of facial regions: eyes, nose and mouth. The open and filled circles represent mean fixation durations for the infants with low and high shyness, respectively. *P<0.05. Error bars indicate the S.E. of the mean.

Although both the high and low shyness groups spent equal time looking at the strangers’ faces, we wondered whether the same type of looking was occurring, especially with regard to the component facial regions. We defined three areas of interest (AOIs) for the eyes, nose and mouth and conducted a 2 (shyness) × 2 (object) × 3 (facial region) repeated-measures ANOVA that revealed a significant interaction between shyness and facial region (F_2, 294_ = 3.81, P<0.03, η_p_^2^ = 0.03, Fig. 3) (Tables S4 and S5). *Post-hoc* testing (Bonferroni) showed that the group with high shyness looked at the eye regions longer than the group with low shyness (P<0.02), whereas the looking time was not significantly different for the other regions (i.e., the nose and mouth) between the high and low shyness groups (P>0.30 for both cases). Neither a main effect of object (i.e., mother or stranger) nor an interaction between object and shyness was observed, which indicates that the highly shy infants were sensitive to the eye region irrespective of whether the viewed faces were of their mothers or strangers. Importantly, this difference between the high and low shyness groups in the time spent looking at the eye region was observed only when we measured the first fixation duration after the stimulus presentation, and it was not observed when we measured the full fixation duration during the presentation period (10 s) (F_2,294_ = 1.13, P>0.32). This result indicates that shyness is associated with an initial impulse to scan the eyes of others.

All of the infants looked longer at the nose and mouth regions than the eye region (P<0.001 for both cases, *post-hoc* comparisons with a Bonferroni correction). This result is consistent with previous findings that talking faces, which are similar to smiling faces with moving mouths, attract infants’ attention more to the mouth region than to the other regions in the second half of the first year of life [38]. Preferences for the facial region vary with infants’ ages, as 4 m.o. infants prefer eyes over the mouth and 6 m.o. infants prefer both eyes and mouth [39]–[42].

There were no significant differences in facial region preferences with regard to the infants’ age, fear temperament and approach temperament (ANOVA and correlational analysis, Table S6).

### Scanning Patterns with Different Gaze Directions

**Figure 4:**
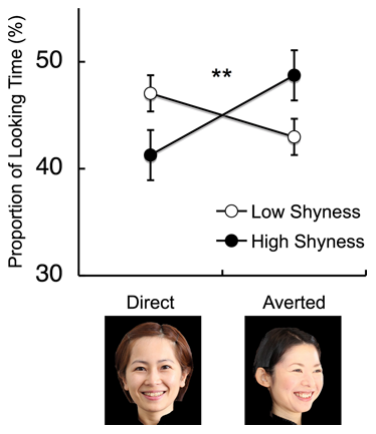
Infants’ visual preferences for different directions of the face and gaze. This figure shows the mean percentile fixation duration for the different types of face stimuli, including strangers’ faces with direct and averted gaze. The bottom pictures are examples of the face stimuli. The open and filled circles represent the mean fixation durations for the infants with low and high shyness, respectively. **P<0.01. Error bars indicate the S.E. of the mean.

We also examined differences in gaze direction preferences between infants with high shyness and those with low shyness. When two strangers, one with a direct gaze and the other with an averted gaze, were presented simultaneously, the infants showed a significant interaction between shyness and gaze direction (F_1, 98_ = 8.14, P<0.01, η_p_^2^ = 0.08, Fig. 4) (Tables S7 and S8). *Post-hoc* testing (Bonferroni) showed that the infants with low shyness looked longer at strangers with a direct gaze than the infants with high shyness (P<0.05), whereas the infants with high shyness looked longer at strangers with an averted gaze than the infants with low shyness (P<0.05).

There were no significant differences in gaze direction preferences with regard to the infants’ age, fear temperament and approach temperament (ANOVA and correlational analysis, Table S9).

## Discussion

This study is the first to show that shy infants possess an approach-avoidance conflict in their temperament. Infant shyness has been viewed as a simpler form of withdrawal, an inhibited approach or a fear of strangers. However, we found that shy infants had a more complex repertoire in that they experienced the seemingly opposing constructs of both high approach and high fear, which has only been observed in children in later developmental stages [13], [14], [16], [17]. We further demonstrated that this conflicted shyness in infancy was associated with an initial hypersensitivity to the eye region, regardless of whether mothers or strangers were fixated on, and with a preference for an averted gaze over a directed gaze when viewing strangers’ faces. Importantly, neither the infants’ age nor their individual temperament (i.e., fear or approach) explained this initial hypersensitivity to the eyes.

At approximately 8 months of age, many infants start reacting shyly to adult strangers, with interindividual differences ranging from extreme shyness to the complete absence of shyness [5]. These differences may be the result of differing thresholds for sympathetic nervous system activation [43], [44]. However, this individual variation in shyness to strangers does not show enough temporal stability over the first 18 months to be considered a stable personality trait [45]. It is only later in development that the shyness trait can first be observed, which has led many researchers to focus on concurrent and predictive correlates [46]–[48]. In contrast, our study focused on the early form of infant shyness, which may be a phenotype present during the developmental process in which infants exhibit an affective state rather than a stable personality trait in socially unfamiliar situations. Thus, the onset and intensity of infant shyness with interindividual differences may reflect developmental changes and thresholds in infants’ neurophysiological responses to strangers, possibly in the amygdala. A human patient with amygdala damage failed to look normally at the eye region when viewing facial expressions [49]. The amygdala also participates in processing information from faces’ eye regions [50]–[52]. Thus, the observed initial hypersensitivity to the eye regions in shy infants may be the result of hyperactivity of the amygdala with a low threshold of response to strangers. Longer looking times toward the eye region in shy infants were observed only during the first fixation to the stimuli. A plausible function of the amygdala is to direct one’s own gaze immediately to the eyes of others and to seek out potential sources of salient social information [49]. We speculate that the initial hypersensitivity and over-seeking of eyes in shy infants may subsequently induce a negative response, such as fear or anxiety, to the direct gaze from a stranger and a preference for the averted gaze. This initial hypersensitivity to eyes may decline with increasing age and with the functional maturation of the emotion regulation systems that are controlled by the prefrontal cortex [53].

As shyness behaviors are induced in real, intense social situations with a heightened arousal level [18], our results from a laboratory setting, in which infants looked at stimulus faces on a monitor, may differ from natural situations. The two-dimensional virtual face stimuli may be insufficient with regard to attention level to induce infants’ shy experiences and subsequent gaze avoidance, and as a result, shy infants may have spent the same amount time looking as infants with low shyness. This possibility suggests that shy infants are more sensitive to human faces, given that they initially increased their time spent looking at the eyes when compared with infants with low shyness.

We previously provided evidence that infants spend *less* time looking at intermediate faces between mothers and strangers than at the prototypes (i.e., the mother’s or stranger’s face) [28]. In this study, we used the intermediate faces to assess how shyness affects infants’ sensitivity to their mothers’ faces. We speculated that if shy infants are sufficiently sensitive to their mothers’ faces, they should prefer their mothers’ faces to the intermediate faces (as observed in typical infants). We also expected that shy infants may prefer intermediate faces to strangers’ faces despite the imperfectness of the hybrid pseudo-mothers’ faces, which would differ from the preference shown by less-shy infants. However, shy infants did not prefer the intermediate faces to the prototypes, which was also observed for the infants with low shyness (Fig. 2). This result indicates that the lower preference for the intermediate faces relative to the prototypes is a robust phenomenon in early infants, irrespective of shyness.

Most work on infant shyness has postulated that this shyness is conceptually identical to a fear of strangers or to behavioral inhibition of the socially unfamiliar, but it is rarely thought to result from social-approach motivation concomitantly with behavioral inhibition. The lack of research regarding this topic may stem from a widely accepted assumption that approach and inhibition reside on two ends of a single continuum. However, evidence from other sources suggests that approach and inhibition may be more appropriately viewed as separate entities. For example, the behavioral approach system [54], the behavioral facilitation system [55] and the expectancy-foraging system [56] describe structures that lead to approach in response to cues or that motivate exploratory activity. Conversely, the harm avoidance dimension [57] and the behavioral inhibition systems [54], [55] halt the appetitive approach to stimuli, which signals punishment or non-reward. Similarly, Kinsbourne [58] asserted that approach was largely controlled by activity in the left hemisphere of the brain, whereas the inhibition of approach was primarily under the influence of the right hemisphere. Furthermore, important findings regarding the dissociated entities of approach and inhibition have been reported in infant studies. Approach and inhibition follow different developmental trajectories, with greater gains in inhibition between 6 and 12 months, whereas approach is relatively stable over this time period [5], [8], [29], [36].

A similar conceptualization of shy subtypes was articulated by Asendorpf [13], [14], who argued that high and low social approach and avoidance lead to different behavioral combinations. For example, individuals who score high on both approach and avoidance are described as shy (or conflicted shy), those who score low on approach and high on avoidance are described as avoidant, those who score low on both approach and avoidance are introverts and those who score high on approach and low on avoidance are sociable. Physiological studies support this classification of shy subtypes, particularly with regard to the difference between conflicted shyness (i.e., high-approach/high-avoidance) and avoidant (i.e., low-approach/high-avoidance) subtypes [59], [60]. Schmidt [59] found that, although participants with conflicted shyness an avoidant participants both exhibited a pattern of greater relative right frontal EEG activity at rest, which is a marker of fear dysregulation [61], the two subtypes were distinguishable based upon their pattern of activity in the left, but not the right, frontal area. The participants with conflicted shyness exhibited significantly higher activity in the left frontal EEG site than the avoidant participants. In addition, the conflicted participants exhibited a significantly faster and more stable heart rate than the avoidant participants in response to an anticipated unfamiliar social situation [60].

Our results are consistent with the previously mentioned studies. Infant shyness is not a single form of behavioral inhibition; rather, it is well explained by the combination of approach and avoidance, i.e., high-approach and high-avoidance temperaments as observed in conflicted shyness. Indeed, infants with high and low fear scores did not show a significant difference in their initial hypersensitivity to the eyes, which could be a psychological marker of shy behavior in children [26] and infants. An interesting aspect of our research is that infants were exposed to only positive facial expressions. Thus, the infant’s approach-avoidance conflict appeared even in the presence of positive emotions by strangers without neutral or negative emotions.

## Supporting Information

Figure S1 **Cross-sectional depiction of the relationship between infant age and shyness scores.** Shyness scores are plotted as a function of infant age in months. The solid line represents a regression line of the distribution. No obvious relation was found between shyness and infant age. R: correlation coefficient.
(PDF)

Table S1 **Descriptive statistics for** **Fig. 2** **.**
(PDF)

Table S2 **Result of two-way ANOVA for** **Fig. 2** **.**
(PDF)

Table S3 **Results of ANOVA/correlational analysis for infant’s characteristics and face preference (related to** **Fig. 2** **).**
(PDF)

Table S4 **Descriptive statistics for** **Fig. 3** **.**
(PDF)

Table S5 **Result of three-way ANOVA for** **Fig. 3** **.**
(PDF)

Table S6 **Results of ANOVA/correlational analysis for infant’s characteristics and facial region preference (related to** **Fig. 3** **).**
(PDF)

Table S7 **Descriptive statistics for** **Fig. 4** **.**
(PDF)

Table S8 **Result of two-way ANOVA for** **Fig. 4** **.**
(PDF)

Table S9 **Results of ANOVA/correlational analysis for infant’s characteristics and gaze/face direction preference (related to** **Fig. 4** **).**
(PDF)
