# Supplementary material for: Shyness in Early Infancy: Approach-Avoidance Conflicts in Temperament and Hypersensitivity to Eyes during Initial Gazes to Faces
Source: PLoS One. 2013 Jun 5;8(6):e65476. doi: 10.1371/journal.pone.0065476 (PMC3673991; doi:10.1371/journal.pone.0065476)
Supplement: Figure S1 — Cross-sectional depiction of the relationship between infant age and shyness scores. Shyness scores are plotted as a function of infant age in months. The solid line represents a regression line of the distribution. No obvious relation was found between shyness and infant age. R: correlation coefficient. (PDF) [file pone.0065476.s001.pdf]

Supporting Information

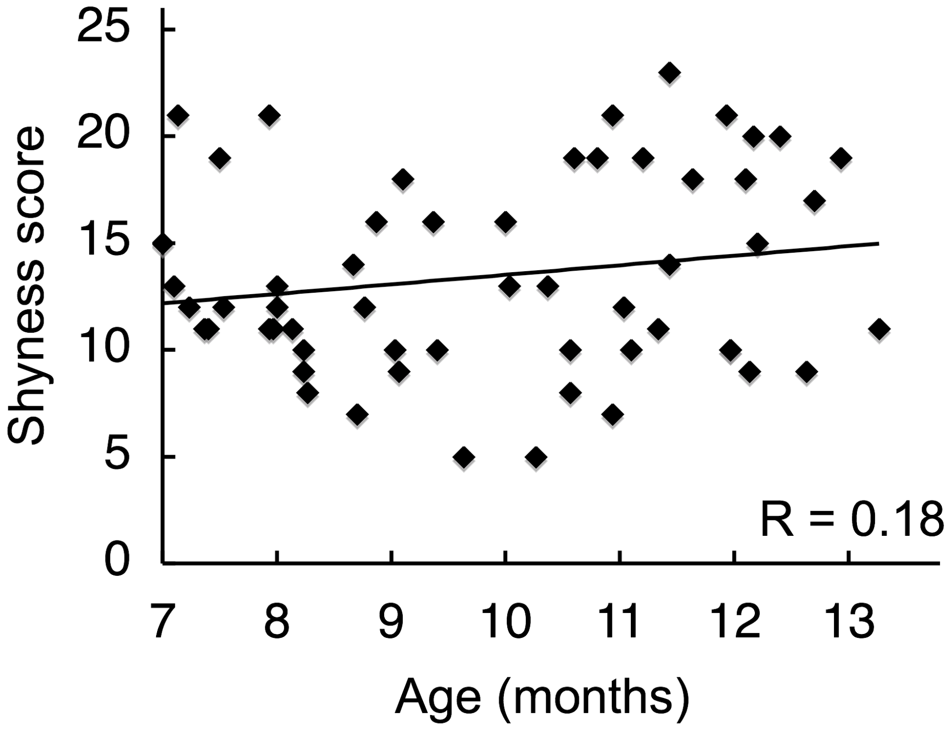

Figure S1. Cross-sectional depiction of the relationship between infant age and shyness scores.

Shyness scores are plotted as a function of infant age in months. The solid line represents a regression line of the distribution. No obvious relation was found between shyness and infant age. R: correlation coefficient.
