# Supplementary material for: Shyness in Early Infancy: Approach-Avoidance Conflicts in Temperament and Hypersensitivity to Eyes during Initial Gazes to Faces
Source: PLoS One. 2013 Jun 5;8(6):e65476. doi: 10.1371/journal.pone.0065476 (PMC3673991; doi:10.1371/journal.pone.0065476)
Supplement: Table S1 — Descriptive statistics for Fig. 2 . (PDF) [file pone.0065476.s002.pdf]

Table S1. Descriptive statistics for Fig.2

		Low shyness			High shyness	
		Mother	Intermediate	Stranger	Mother	Intermediate	Stranger
Number		34	34	34	17	17	17
Mean		37.37	32.52	35.29	37.00	29.86	38.10
Std. Error of Mean		0.94	1.00	0.93	1.26	1.44	1.33
Median		36.46	31.32	35.56	37.06	28.74	39.52
Std. Deviation		5.51	5.81	5.41	5.18	5.92	5.49
Variance		30.40	33.76	29.23	26.81	35.08	30.13
Skewness		0.87	0.26	0.03	-0.09	0.24	-0.10
Std. Error of Skewness		0.40	0.40	0.40	0.55	0.55	0.55
Kurtosis		1.70	-0.19	-0.11	-0.62	0.35	-0.61
Std. Error of Kurtosis		0.79	0.79	0.79	1.06	1.06	1.06
Range		25.93	22.38	22.87	17.93	23.21	19.93
Percentile	25	34.47	29.96	31.62	33.19	26.47	33.74
	50	36.46	31.33	35.56	37.06	28.74	39.52
	75	40.04	35.40	38.50	41.58	33.40	41.46
