# Supplementary material for: Shyness in Early Infancy: Approach-Avoidance Conflicts in Temperament and Hypersensitivity to Eyes during Initial Gazes to Faces
Source: PLoS One. 2013 Jun 5;8(6):e65476. doi: 10.1371/journal.pone.0065476 (PMC3673991; doi:10.1371/journal.pone.0065476)
Supplement: Table S2 — Result of two-way ANOVA for Fig. 2 . (PDF) [file pone.0065476.s003.pdf]

Table S2. Result of two-way ANOVA for Fig.2

Source	Type III Sum of Squares	df	Mean Square	F	Sig.	Partial Eta Squared
Corrected Model	1083.03[a]	5	216.61	6.99	0.00	0.19
Intercept	166836.77	1	166836.77	5384.83	0.00	0.97
Shyness	0.18	1	0.18	0.01	0.94	0.00
Object	1004.71	2	502.36	16.21	***0.00	0.18
Shyness * Object	171.14	2	85.57	2.76	0.07	0.04
Error	4554.47	147	30.98			
Total	193460.33	153				
Corrected Total	5637.49	152				

Shyness = {Low, High}, Object = {Mother, Intermediate, Stranger}

Sig.: Significance Probability, df: Degrees of Freedom, ***P<0.001

a) R Squared = 0.19 (Corrected R Squared = 0.17)
