# Supplementary material for: Shyness in Early Infancy: Approach-Avoidance Conflicts in Temperament and Hypersensitivity to Eyes during Initial Gazes to Faces
Source: PLoS One. 2013 Jun 5;8(6):e65476. doi: 10.1371/journal.pone.0065476 (PMC3673991; doi:10.1371/journal.pone.0065476)
Supplement: Table S3 — Results of ANOVA/correlational analysis for infant’s characteristics and face preference (related to Fig. 2 ). (PDF) [file pone.0065476.s004.pdf]

Table S3. Results of ANOVA/correlational analysis for infant's characteristics and face preference (related to Fig.2)

Characteristic (Temperament or Age)	Interaction with Object (ANOVA)			Correlation with Preference of Mothers [a)]		
	N	$F_{1,98}$	Sig.	R	t_{49}	Sig.
Shyness	34 (Low), 17 (High)	1.94	0.17	-0.09	-0.65	0.52
Fear	32 (Low), 19 (High)	0.66	0.42	-0.03	-0.20	0.84
Approach	18 (Low), 33 (High)	0.37	0.55	0.11	0.76	0.45
Age	26 (Young), 25 (Old)	2.11	0.15	0.16	1.17	0.25

Object = {Mother, Stranger}, R: Correlation Coefficient, Sig.: Significance Probability

a) Correlational analysis of looking time of mother's face as a function of age or temperament's scores.
