# Supplementary material for: Shyness in Early Infancy: Approach-Avoidance Conflicts in Temperament and Hypersensitivity to Eyes during Initial Gazes to Faces
Source: PLoS One. 2013 Jun 5;8(6):e65476. doi: 10.1371/journal.pone.0065476 (PMC3673991; doi:10.1371/journal.pone.0065476)
Supplement: Table S4 — Descriptive statistics for Fig. 3 . (PDF) [file pone.0065476.s005.pdf]

Table S4. Descriptive statistics for Fig. 3

		Low shyness			High shyness		
		Eyes	Nose	Mouth	Eyes	Nose	Mouth
Number		34	34	34	17	17	17
Mean		11.44	44.64	38.01	21.59	39.09	34.44
Std. Error of Mean		2.08	3.19	3.78	3.81	3.89	5.27
Median		8.10	45.12	37.19	20.69	36.01	39.57
Std. Deviation		12.15	18.61	22.05	15.70	16.06	21.74
Variance		147.73	346.23	485.99	246.43	257.77	472.54
Skewness		0.85	-0.38	0.45	0.42	-0.04	-0.75
Std. Error of Skewness		0.40	0.40	0.40	0.55	0.55	0.55
Kurtosis		-0.35	-0.45	-0.42	-0.60	-0.19	-0.90
Std. Error of Kurtosis		0.79	0.79	0.79	1.06	1.06	1.06
Range		37.93	72.74	90.00	54.00	58.99	58.54
Percentile	25	0.00	34.90	22.07	8.89	31.63	13.54
	50	8.10	45.12	37.19	20.69	36.01	39.57
	75	19.27	63.13	53.67	33.84	51.88	53.40
