# Supplementary material for: Shyness in Early Infancy: Approach-Avoidance Conflicts in Temperament and Hypersensitivity to Eyes during Initial Gazes to Faces
Source: PLoS One. 2013 Jun 5;8(6):e65476. doi: 10.1371/journal.pone.0065476 (PMC3673991; doi:10.1371/journal.pone.0065476)
Supplement: Table S5 — Result of three-way ANOVA for Fig. 3 . (PDF) [file pone.0065476.s006.pdf]

Table S5. Result of three-way ANOVA for Fig.3

Source	Type III Sum of Squares	df	Mean Square	F	Sig.	Partial Eta Squared
Corrected Model	49719.37[a]	11	4519.94	10.40	0.00	0.28
Intercept	270540.89	1	270540.89	622.73	0.00	0.68
Shyness	8.06	1	8.06	0.02	0.89	0.00
Object	26.76	1	26.76	0.06	0.80	0.00
Region	32112.21	2	16056.11	36.96	***0.00	0.20
Shyness * Object	29.46	1	29.46	0.07	0.80	0.00
Object * Region	1130.13	2	565.06	1.30	0.27	0.01
Shyness * Region	3312.74	2	1656.37	3.81	*0.02	0.03
Shyness * Object * Region	410.74	2	205.37	0.47	0.62	0.00
Error	127726.56	294	434.44			
Total	480697.86	306				
Corrected Total	177445.94	305				

Shyness = {Low, High}, Object = {Mother, Stranger}, Region = {Eyes, Nose, Mouth}

Sig.: Significance Probability, df: Degrees of Freedom, *P<0.05, ***P<0.001

a) R Squared = 0.28 (Corrected R Squared = 0.25)
