# Supplementary material for: Shyness in Early Infancy: Approach-Avoidance Conflicts in Temperament and Hypersensitivity to Eyes during Initial Gazes to Faces
Source: PLoS One. 2013 Jun 5;8(6):e65476. doi: 10.1371/journal.pone.0065476 (PMC3673991; doi:10.1371/journal.pone.0065476)
Supplement: Table S6 — Results of ANOVA/correlational analysis for infant’s characteristics and facial region preference (related to Fig. 3 ). (PDF) [file pone.0065476.s007.pdf]

Table S6. Results of ANOVA/correlational analysis for infant's characteristics and facial region preference (related to Fig.3)

Characteristic (Temperament or Age)	Interaction with Facial Region (ANOVA)			Correlation with Preference of Eyes [a)]		
	N	$F_{2,294}$	Sig.	R	t_{49}	Sig.
Shyness	34 (Low), 17 (High)	3.81	*0.03	0.29	2.09	*0.04
Fear	32 (Low), 19 (High)	1.28	0.26	0.18	1.30	0.20
Approach	18 (Low), 33 (High)	0.06	0.81	0.00	0.03	0.98
Age	26 (Young), 25 (Old)	0.22	0.64	-0.23	-1.68	0.10

Facial Region = {Eyes, Nose, Mouth}, R: Correlation Coefficient, Sig.: Significance Probability, *P<0.05

a) Correlational analysis of looking time of eyes' region as a function of age or temperament's scores.
