# Supplementary material for: Shyness in Early Infancy: Approach-Avoidance Conflicts in Temperament and Hypersensitivity to Eyes during Initial Gazes to Faces
Source: PLoS One. 2013 Jun 5;8(6):e65476. doi: 10.1371/journal.pone.0065476 (PMC3673991; doi:10.1371/journal.pone.0065476)
Supplement: Table S7 — Descriptive statistics for Fig. 4 . (PDF) [file pone.0065476.s008.pdf]

Table S7. Descriptive statistics for Fig. 4

		Low shyness		High shyness	
		Direct	Averted	Direct	Averted
Number		34	34	17	17
Mean		47.04	42.96	41.27	48.73
Std. Error of Mean		1.69	1.69	2.35	2.35
Median		46.73	43.27	40.83	49.17
Std. Deviation		9.88	9.88	9.39	9.39
Variance		97.56	97.56	88.11	88.11
Skewness		-0.21	0.21	0.27	-0.27
Std. Error of Skewness		0.40	0.40	0.56	0.56
Kurtosis		-0.90	-0.90	-1.27	-1.27
Std. Error of Kurtosis		0.79	0.79	1.09	1.09
Range		37.39	37.39	27.91	27.91
Percentile	25	38.95	34.56	32.20	41.96
	50	46.73	43.27	40.83	49.17
	75	55.44	51.05	48.04	57.80
