# Supplementary material for: Shyness in Early Infancy: Approach-Avoidance Conflicts in Temperament and Hypersensitivity to Eyes during Initial Gazes to Faces
Source: PLoS One. 2013 Jun 5;8(6):e65476. doi: 10.1371/journal.pone.0065476 (PMC3673991; doi:10.1371/journal.pone.0065476)
Supplement: Table S8 — Result of two-way ANOVA for Fig. 4 . (PDF) [file pone.0065476.s009.pdf]

Table S8. Result of two-way ANOVA for Fig.4

Source	Type III Sum of Squares	df	Mean Square	F	Sig.	Partial Eta Squared
Corrected Model	755.74[a]	3	251.91	2.72	0.05	0.08
Intercept	183600.00	1	183600.00	1981.10	0.00	0.95
Shyness	0.00	1	0.00	0.00	1.00	0.00
Direction	64.71	1	64.71	0.70	0.41	0.01
Shyness * Direction	754.35	1	754.35	8.14	**0.01	0.08
Error	9082.21	98	92.68			
Total	216387.95	102				
Corrected Total	9837.95	101				

Shyness = {Low, High}, Direction = {Direct, Averted}

Sig.: Significance Probability, df: Degrees of Freedom, **P<0.01

a) R Squared = 0.08 (Corrected R Squared = 0.05)
