# Supplementary material for: Shyness in Early Infancy: Approach-Avoidance Conflicts in Temperament and Hypersensitivity to Eyes during Initial Gazes to Faces
Source: PLoS One. 2013 Jun 5;8(6):e65476. doi: 10.1371/journal.pone.0065476 (PMC3673991; doi:10.1371/journal.pone.0065476)
Supplement: Table S9 — Results of ANOVA/correlational analysis for infant’s characteristics and gaze/face direction preference (related to Fig. 4 ). (PDF) [file pone.0065476.s010.pdf]

Table S9. Results of ANOVA/correlational analysis for infant's characteristics and gaze/face direction preference (related to Fig.4)

Characteristic (Temperament or Age)	Interaction with Gaze/Face Direction (ANOVA)			Correlation with Preference of Averted Gaze/Face [a)]		
	N	$F_{1,98}$	Sig.	R	t_{49}	Sig.
Shyness	34 (Low), 17 (High)	8.14	**0.01	0.09	0.64	0.53
Fear	32 (Low), 19 (High)	0.66	0.42	0.18	1.20	0.24
Approach	18 (Low), 33 (High)	0.22	0.64	0.07	0.50	0.62
Age	26 (Young), 25 (Old)	0.14	0.71	0.17	1.18	0.24

Gaze/Face Direction = {Direct, Averted}, R: Correlation Coefficient, Sig.: Significance Probability, **P<0.01

a) Correlational analysis of looking time of averted gaze/face as a function of age or temperament's scores.
